# Comparative Evaluation of Four Extraction Methods of Antioxidant Compounds from *Decatropis bicolor* in Aqueous Medium Applying Response Surface Design

**DOI:** 10.3390/molecules26041042

**Published:** 2021-02-17

**Authors:** Judith Jaimez-Ordaz, Elizabeth Contreras-López, Tania Hernández-Sánchez, Luis Guillermo González-Olivares, Javier Añorve-Morga, Juan Ramírez-Godínez

**Affiliations:** 1 Área Académica de Química, Instituto de Ciencias Básicas e Ingeniería, Universidad Autónoma del Estado de Hidalgo, Mineral de la Reforma, Hidalgo 42184, Mexico; jjaimez@uaeh.edu.mx (J.J.-O.); elizac@uaeh.edu.mx (E.C.-L.); lgonzales@uaeh.edu.mx (L.G.G.-O.); jmorga@uaeh.edu.mx (J.A.-M.); 2 Área Académica de Gastronomía, Instituto de Ciencias Económico Administrativas, Universidad Autónoma del Estado de Hidalgo, Circuito La Concepción Km 2.5 Col. San Juan Tilcuautla, San Agustín Tlaxiaca, Hidalgo 42160, Mexico; tania.hernandez@uaeh.edu.mx

**Keywords:** *Decatropis bicolor*, antioxidants, phenolics, extraction methods

## Abstract

The objective of this paper is to compare conventional, ultrasound, microwave, and French press methods for the extraction of antioxidant compounds from *Decatropis bicolor* in an aqueous medium. This plant is widely used in Mexican traditional medicine for breast cancer treatment. Despite that, there are few studies on *D. bicolor*. Two response surface designs were applied to establish the best conditions of the liberation of antioxidants from *D. bicolor*, which were determined by DPPH• and Ferric Reducing Antioxidant Power (FRAP) techniques. The total phenolic content was evaluated by the Folin-Ciocalteu method. The results showed that *D. bicolor* is a source of antioxidants (669–2128 mg ET/100 g and 553–1920 mg EFe^2+^/100 g, respectively) and phenolic compounds (2232–9929 mg EGA/100 g). Among the physical factors that were analyzed, the temperature was the determinant factor to liberate the compounds of interest by using low concentrations of the sample and short times of extraction. The French press was the most efficient method, obtaining values of antioxidant activity and phenolic compounds even higher than those reported by using extraction methods with solvents such as methanol.

## 1. Introduction

Medicinal plants are used as a source of natural compounds that have a positive impact on health [1,2]. One plant widely used in traditional medicine is *Decatropis bicolor*, belonging to the Rutaceae family. This plant is a 2–3-m tall shrub with small white flowers that is distributed from Mexico to Centroamerica and it is commonly known as arantho, arandho, aranto, golden leaf, among others. In several communities of the state of Hidalgo (Mexico), one of the most common uses of *D. bicolor* is in infusions prepared by boiling the aerial parts in water. This preparation is drunk as a treatment against breast cancer [3]. Other properties reported for this plant include antifungal [4] and anti-inflammatory [5] activities which can be related to the presence of phenolic and antioxidant compounds. 

Phenolics are widely distributed in the plant kingdom and are the main secondary metabolites of plants and herbs. Currently, more than 8000 phenolic structures are known, including simple molecules such as phenolic acids and highly polymerized compounds such as tannins [6,7,8]. Even with this wide structural variety, generally, this group of compounds is often referred to as polyphenols [6]. Of these, phenolic acids, flavonoids, and tannins are considered as the main dietary naturally occurring phenolic compounds [9].

The selection of the proper extraction method of bioactive compounds, such as antioxidant and phenolic compounds, from plants needs meticulous evaluation to get the highest yield as well as to assure the preservation of the beneficial properties of the compounds of interest [2]. Due to the variation in the chemical structure of bioactive compounds present in plants, it is challenging to choose a single method for their extraction [1]. The most common methods used for the extraction of bioactive compounds from vegetal matrixes (plants, vegetables, fruits, and by-products), are maceration, Soxhlet [1,2], and conventional solid-liquid extraction [10], although shaking and heated reflux have also been used [1,10]. Other methods as microwave, microwave-assisted [11,12,13,14], ultrasound-assisted [1,2,14,15,16,17,18], and super-critical fluid extraction [1,2,14,18] have been used to increase extraction yield [2]. The efficiency of the extraction depends on several factors such as temperature, sample (the type of plant and pre-conditioning), time, and the solvent agent, among others. Methanol and ethanol, either pure or in aqueous mixtures, have been the most extensively used agents for the extraction of bioactive compounds from natural sources, mainly plants and plant-based foods [1,10].

Since medicinal plants are used mainly as infusions the use of water-based methods would be of interest for recovering bioactive compounds from these matrixes. The French press is a simple, low-cost, water-based extraction method traditionally used worldwide for coffee preparation. Compared to other methods, it allows better extraction of the soluble compounds present in coffee due to the physical and chemical interactions occurring between the sample and hot water [19,20,21]. Due to this, it is interesting to apply this method for the extraction of bioactive constituents from other sources such as medicinal plants. Therefore, this research aimed at comparing the effect of the extraction method (conventional, ultrasound, microwave, and French press) in an aqueous solution on the liberation of antioxidant compounds and total phenolics of *D. bicolor*. For that, the experiment designs Box-Behnken and Central Composite were applied to determine the best physical conditions to liberate the compounds of interest.

## 2. Results and Discussion

### 2.1. Design of Experiments and Factors Affecting the Liberation of Phenolic and Antioxidant Compounds of D. bicolor in an Aqueous Medium

The polynomials obtained from the experimental designs applied are presented in Table 1. For the extraction of the antioxidant and total phenolic compounds from *D. bicolor* by the conventional and ultrasonic methods, the correlation coefficients determined in the polynomial were superior to 95% for total phenolics and Ferric Reducing Antioxidant Power (FRAP) and higher than 90% for DPPH•. The correlation coefficients for extraction through microwave were R^2^ = 0.99, while for the French press were R^2^ ≥ 0.85. This suggests that the applied designs were appropriate for a reliability level of 95%.

**Figure 1:**
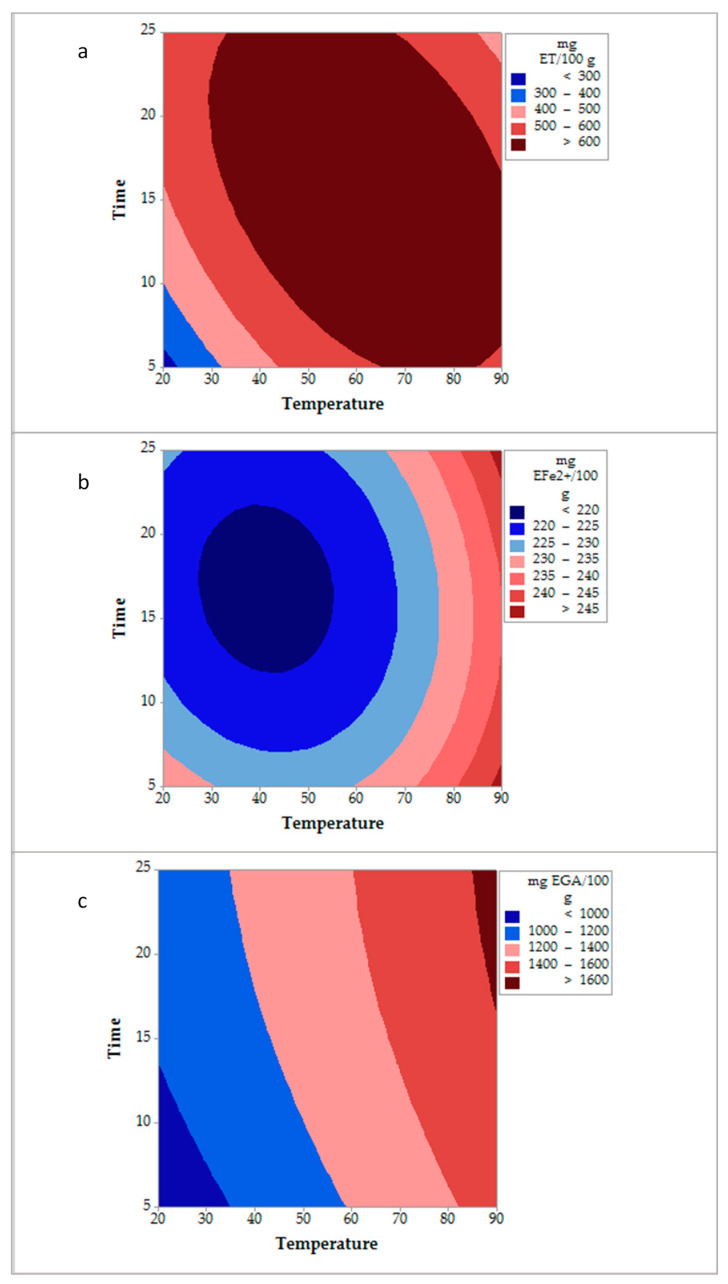
Contour plots obtained for the conventional extraction method of *D. bicolor.* (**a**): DPPH• method, (**b**): Ferric Reducing Antioxidant Power (FRAP) method, and (**c**): Total phenolic compounds.

**Figure 2:**
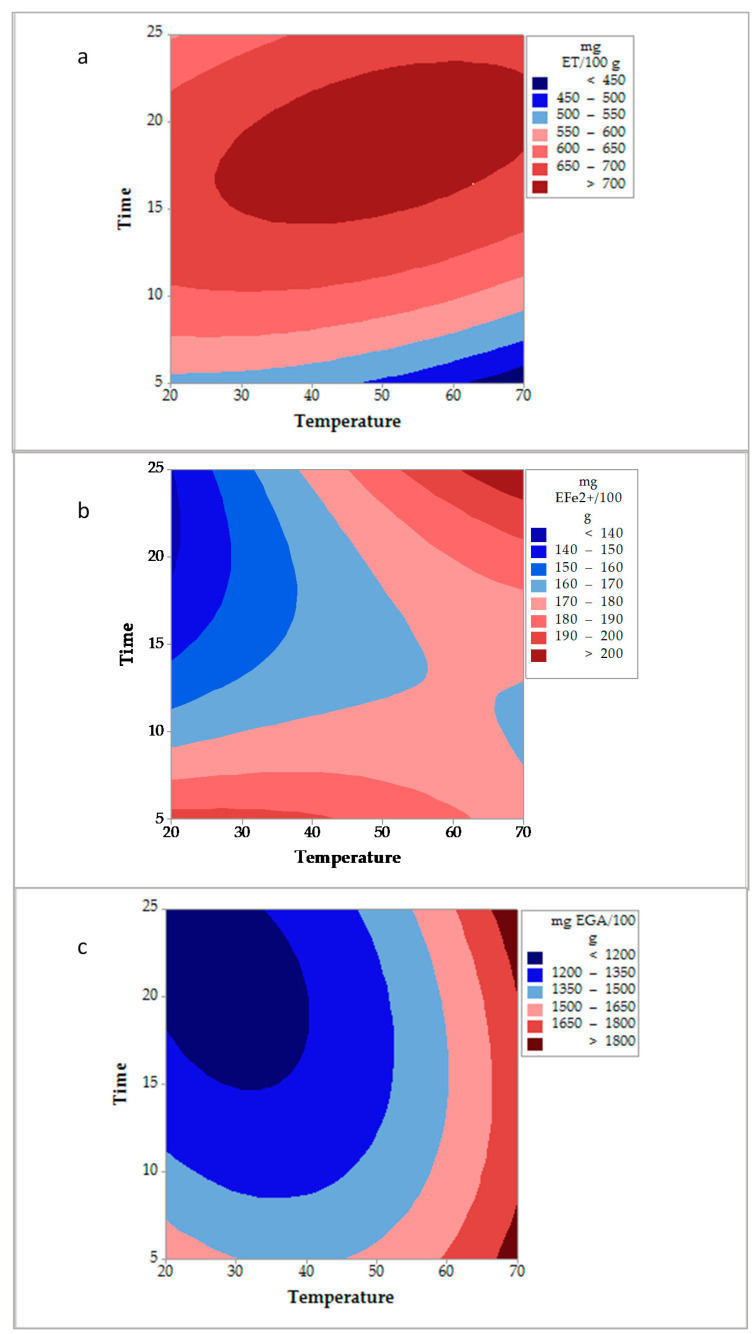
Contour plots obtained for the ultrasound-assisted extraction of *D. bicolor.* (**a**): DPPH• method, (**b**): FRAP method, and (**c**): Total phenolic compounds.

The contour plots obtained from the Box-Behnken experimental design of the conventional and ultrasound-assisted extraction of *D. bicolor* are shown in Figure 1 and Figure 2, respectively. For these extraction methods, the interaction temperature and time (Table 1), using a low amount of sample, influenced the liberation of both antioxidant (via radical and redox) and total phenolic compounds. 

During the conventional extraction, time and temperature were determinant for the liberation of antioxidant compounds. It was observed that low temperatures (40 °C) combined with extraction times above 10 min allowed antioxidant activity values equal or superior to 600 mg ET/100 g (Figure 1a). In the same way, this figure shows that increasing temperature over 65 °C, it is possible to get the same extraction value from 5 min of extraction. On the other hand, by increasing the temperature over 85 °C, antioxidant activity values higher than 245 mg EFe^2+^/100 g were obtained regardless of the time of extraction applied (Figure 1b). In the same way, a higher liberation of total phenolics (≥1650 mg EGA/100 g) was obtained at a temperature of 85 °C and a time of extraction above 15 min (Figure 1c). 

Regarding ultrasound-assisted extraction (Figure 2), it was observed that temperatures from 25 °C and from 10 min of extraction allowed the obtention of antioxidant activity values higher than 650 mg ET/100 g (Figure 2a). During the extraction using the FRAP technique (Figure 2b), longer times of extraction (20–25 min), and higher temperatures (60–70 °C) were needed for the obtention of values of antioxidant activity from 170 to >200 mg EFe^2+^/100 g. Related to total phenolics content (Figure 2c), the highest liberation (values higher than 1800 mg EGA/100 g) occurred at temperatures between 67 and 70 °C regardless of the time of extraction applied. 

**Figure 3:**
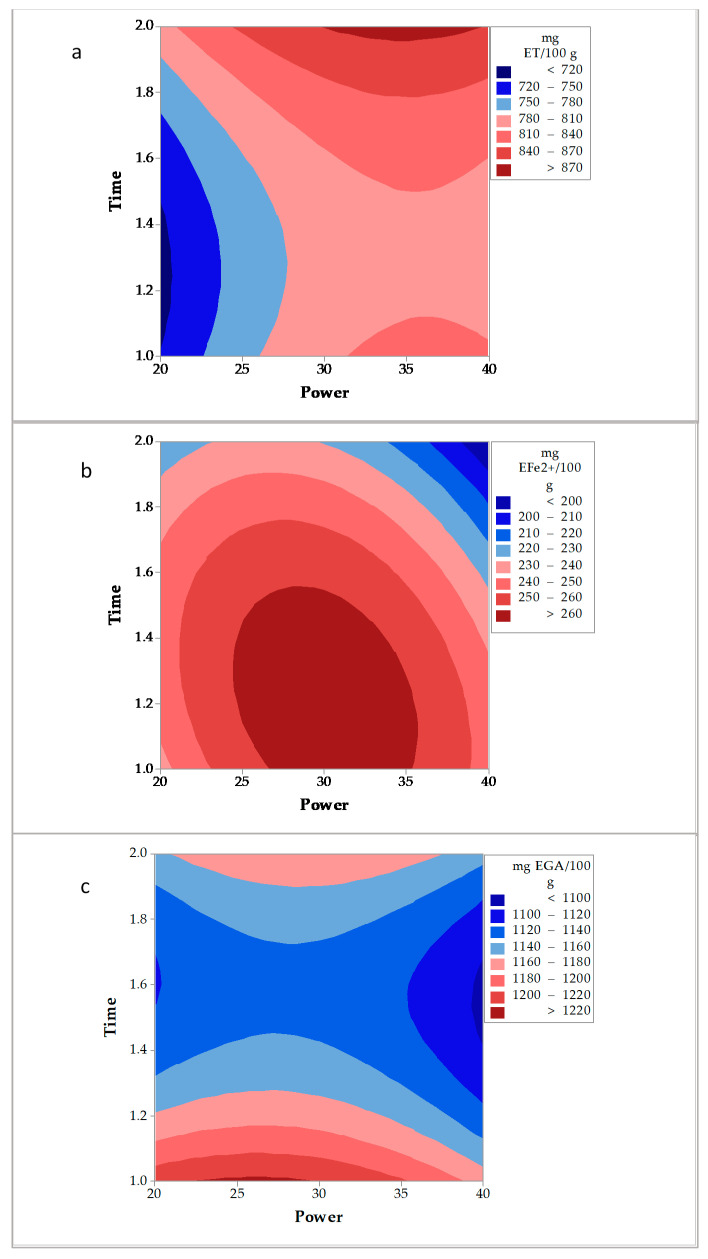
Contour plots for the microwave extraction of D. bicolor. (**a**): DPPH• method, (**b**): FRAP method, and (**c**): Total phenolic compounds.

By using the microwave extraction (Figure 3), the contour plots obtained from the Box-Behnken design (BBD) showed that interaction power and time, using a low amount of sample (2 g) were the main factors affecting the extraction (Table 1). For the DPPH technique (Figure 3a), it was observed that applying a time of exposition close to 2 min and power between 30 and 40, values higher than 870 mg ET/100 g were obtained. With this method, the use of low power (25%–35%) and a short time of extraction (1–2 min) allowed the highest values of antioxidant activity (>260 mg EFe^2+^/100 g) (Figure 3b) and phenolic compounds (>1220 mg EGA/100 g) (Figure 3c). 

**Figure 4:**
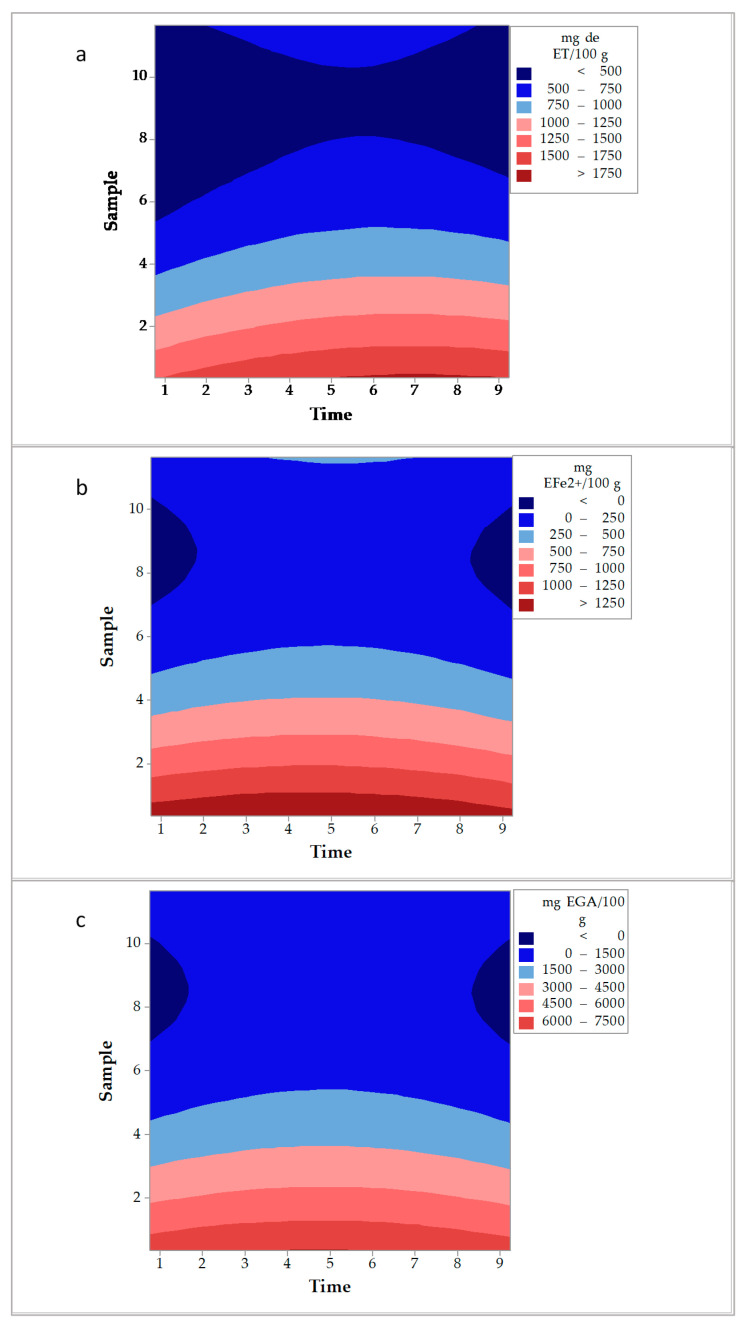
Contour plots obtained for the extraction of *D. bicolor* with a French press. (**a**): DPPH• method, (**b**): FRAP method, and (**c**): Total phenolic compounds.

Regarding the extraction carried out using a French press, the contour plots obtained from the Central Composite design (DCC) (Figure 4) proved that the efficiency of this method was superior to the other methods tested. Using a French press, the extraction time and the amount of sample had a significant effect on the efficiency observed. In this case, the lowest amount of *D. bicolor* was needed to obtain the highest values of antioxidant activity (1750 mg ET/100 g and 1250 mg EFe^2+^/100 g, Figure 4a,b, respectively) and phenolic compounds (7500 mg EGA/100 g, Figure 4c).

### 2.2. Measures of the Response Variables

The response variables measured (antioxidant activity and phenolic compounds) based on the 58 assays obtained from the applied experimental designs through the different extraction methods compared are showed in Table 2. The higher values of antioxidant activity and total phenolics were in the following order: French press > microwave > ultrasound > conventional.

There is a lack of information in the literature about the antioxidant activity and phenolic compounds from *D. bicolor* as well as the extraction of those compounds from plant matrixes in an aqueous media. However, our results are comparable to those reported for other plants (*Limonium sinuatum*, *Thymus serpyllum* L., *Thymus algeriensis*, *Thymus vulgaris, Lycium ruthenicum, S. miltiorrhiza Bge., P. multiflorum Thunb. (Stem), R. sacra Fu, S. cuneata Rehd. et Wils., F. rhynchophylla Hance, P. persica (Linn) Batsch., C. foetida L., P. lactiflora Pall., T. farfara L., and S. officinalis L*. among others) extracted through different methods [22,23,24,25,26,27] in which authors determined values of antioxidant activity via radical scavenging from 305 to 1544 mg ET/100 g, via redox from 107 to 1432 mg EFe^2+^/100 g and total phenolics from 523 to 4730 mg EGA/100 g. The large variability of the published results can be explained due to differences in the plant species analyzed and the methods and conditions used, among other factors [23,24,25,26,27,28,29].

Regarding conventional extraction, this method has been extensively used worldwide for the liberation of compounds of interest from plants and other food matrixes [30,31]. However, the use of solvents, mainly methanol and ethanol, could be a disadvantage compared to green extraction methods such as microwave and ultrasound [30,31]. In our research, we used water as a sole extractant agent, simulating conditions for the elaboration of an infusion. The observed results (Table 2) indicate that for conventional extraction in an aqueous media, it is necessary to use high temperatures (90 °C) to reach the highest liberation of compounds from *D. bicolor*. This agrees with the observed for the liberation of antioxidant and total phenolic compounds from *Zingiber officinale* and *Melissa officinalis* [32,33].

Related to ultrasound-assisted extraction, we found that the temperature was a determinant factor in the liberation of antioxidant and phenolic compounds (Table 2). It was observed that increasing temperature up to 70 °C allowed the highest liberation of antioxidants from *D. bicolor*. This is in agreement with data reported for antioxidants extracted from different vegetable matrixes [34,35,36,37]. Similarly, Contreras-López et al. [38] used thermo-ultrasound, applying temperatures from 30 to near 60 °C to obtain the higher liberation of antioxidant and phenolic compounds from ginger (*Zingiber officinalis*). In our research, it was also observed that the best results were obtained by using times of extraction no longer than 15 min. This agrees with the report by Albu et al. [39] for extracts of *Rosmarinus officinalis*. Some studies applying ultrasound have shown that the use of short times of extraction prevents the sample from being exposed to conditions that might affect or degrade the compounds of interest. In this sense, Xu et al. [22], observed the degradation of phenolic compounds by increasing the sonication time of *Limonium sinuatum* over 10 min.

Microwave has been used to extract antioxidant compounds in a great variety of vegetable food matrices [40,41,42]. Concerning *D. bicolor*, using this method, the highest values of antioxidant activity (by DPPH and FRAP) and total phenolics were obtained by using 2% sample, 30% power, and short times of extraction (1–2 min) (Table 2). Several authors reported that the optimal time of exposure in microwave goes from 0.5–1 min [41,43,44]. This has also been proven for the extraction of antioxidant compounds from leaves of *Pistacia lentiscus* getting a significant increase using 1 min of extraction, while a reduction was observed using 2 min [44]. Furthermore, using the microwave technique, a greater antioxidant capacity has been observed in extracts from medicinal plants like *Eucommia ulmoides*, *Terminalia bellerica*, *Artemisia sphaerocephala*, *Pistacia lentiscus,* and *Prunus laurocerasus*, compared to the conventional and ultrasound-assisted extraction [43,45,46,47,48]. Unlike other techniques, by using the microwave technique, heating is more efficient since the vibration of molecules happens directly in the middle, which contributes to make it the most efficient and fast extraction technique [12,42].

Related to the French press, the highest values of antioxidant capacity and total phenolics were obtained using the lowest amount of sample (0.34%), after 5 min of extraction (Table 2). This allows us to say that for *D. bicolor*, the French press was the most efficient of the four extraction methods analyzed. This could be explained because the pressure is applied uniformly to the sample [49]. The French press has been extensively used for the preparation of coffee worldwide, allowing the obtention of extracts with a high antioxidant capacity, due to the presence of hydrophilic as well as phenolic (mono- and dicaffeoylquinic acids) and non-phenolic compounds [14,15,16,50,51,52]. The use of the French press has been also efficient for obtaining higher extraction rates of fatty acids from coffee compared with conventional extraction [20,53].

The values of antioxidant capacity and total phenolics obtained for *D. bicolor* through a French press (434–2128 mg ET/100 g and 110–1920 mgEFe^2+^/100 g, 684–9929 mg EGA/100 g) are superior to those reported about the extraction of bioactive compounds from several vegetal matrixes including flowers [22], tea [36], medicinal and aromatic plants [23,27,30,38], and agri-food by-products [25,41]. Those studies include the use of different extraction methods (i.e., conventional, microwave, and ultrasound) and involve several extractant agents such as water, methanol, ethanol, acetone, ethyl acetate as well as their mixtures with water.

### 2.3. Validation of the Experimental Designs

The accuracy of the extraction model was checked through the validation of the experimental designs. It was observed that experimental results of antioxidant compounds and total phenolics from *D. bicolor* in an aqueous media were similar to predictive values.

#### 2.3.1. Conventional Extraction

The optimal conditions for the conventional extraction were 90 °C, 17 min, and 2% of the sample. The model was validated by comparing the predicted (2244.5 mg EGA/100 g, 1518.9 mg ET/100 g, and 699.5 mg EFe^2+^/100 g) and experimental values of phenolic (2242 mg EGA/100 g) and antioxidant compounds (1521 mg ET/100 g for DPPH• and 697 mg EFe^2+^/100 g). No significant differences were found (*p* > 0.05).

#### 2.3.2. Ultrasound-Assisted Extraction

The best conditions for the extraction of antioxidant and phenolic compounds from *D. bicolor* using ultrasound-assisted were 70 °C, for 15 min with a sample concentration of 2%. No significant differences (*p* > 0.05) were found by comparing the predicted values (2973.4 mg EGA/100 g of total phenolics and for the antioxidant activity of 1215.9 mg ET/100 g for DPPH and 554.9 mg EFe^2+^/100 g for FRAP) and experimental results (2971 mg EGA/100 g of total phenolics and for the antioxidant activity, 1215 mg ET/100 g for DPPH and 553 mg EFe^2+^/100 g for FRAP).

#### 2.3.3. Extraction with Microwave

For the extraction with microwave, the optimal conditions were 30% of power, 2 min, and 2% of sample concentration. No significant differences (*p* > 0.05) were found between the predicted values (3282.3 mg EGA/100 g of total phenolics and for the antioxidant activity of 1516.2 mg ET/100 g for DPPH and 730.9 mg EFe^2+^/100 g for FRAP) and the experimental values (3280 mg EGA/100 g of total phenolics and for the antioxidant activity of 1514 mg ET/100 g for DPPH and 733 mg EFe^2+^/100 g for FRAP).

#### 2.3.4. Extraction with French Press

The extracts of *D. bicolor* obtained using a sample concentration of 0.3% and 5 min of exposure in hot water (90 °C) presented the highest antioxidant activity and phenolic content values. No significant differences were observed (*p* > 0.05) between the predicted (9932 mg EGA/100 g of total phenolics and for the antioxidant activity of 2126 mg ET/100 g for DPPH and 1923.1 mg EFe^2+^/100 g for FRAP) and the experimental values (9929 mg EGA/100 g of total phenolics and for the antioxidant activity of 2128 mg ET/100 g for DPPH and 1920 mg EFe^2+^/100 g for FRAP).

The results obtained from the validation of the experimental designs of the different extraction methods used to liberate bioactive compounds from *D. bicolor* in aqueous media indicate their accuracy and reproducibility. The four extraction methods analyzed in this research proved to be adequate. Nevertheless, each one presents advantages and disadvantages that should be taken into account before been selected. There is a trend in using green technologies to reduce the use of solvents without affecting the efficiency of the extraction. It is worth encouraging research on water-based methods for the extraction of compounds of interest from potential antioxidant sources such as *Decatropis bicolor*.

## 3. Materials and Methods

**Figure 5:**
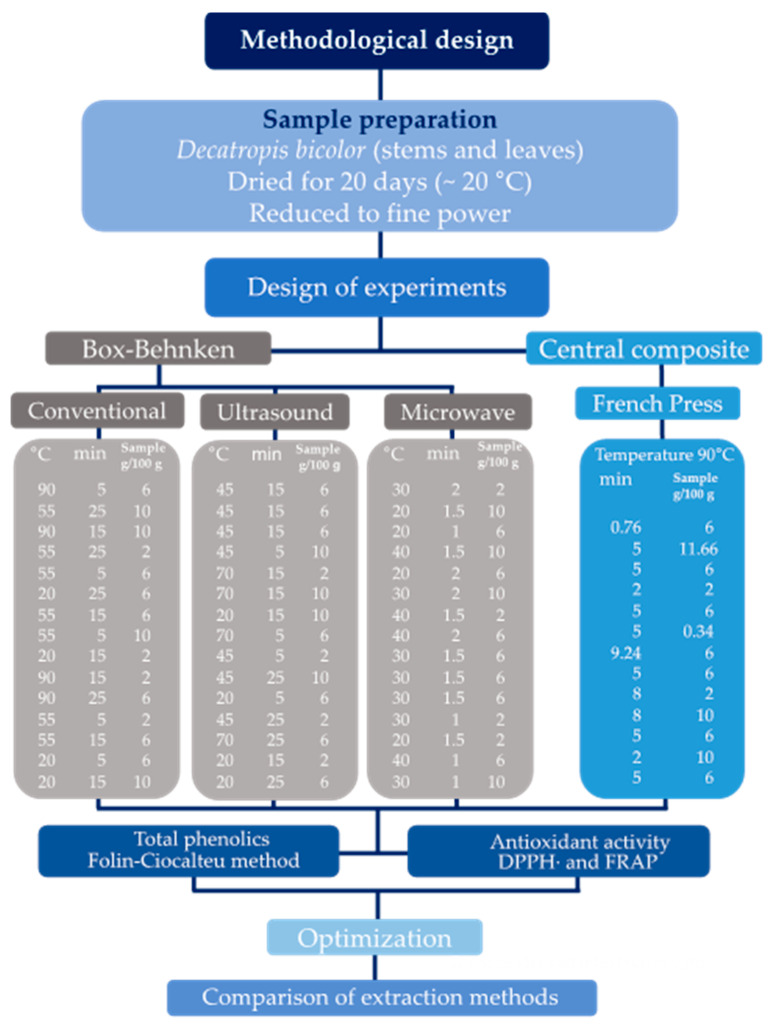
General methodology applied in this research.

Figure 5 shows the general methodology applied for the comparison of four extraction methods of antioxidant compounds from *D. bicolor*.

### 3.1. Materials and Reagents

Folin–Ciocalteu reagent (2N), 2,2-diphenyl-1picrylhydrazyl free radical (DPPH•), (±)-6-hydroxy-2,5,7,8-tetramethylchromane-2-carboxylic acid (Trolox, 97%) were purchased from Sigma-Aldrich (St. Louis, MO, USA). Gallic acid, potassium persulfate, ethanol, anhydrous sodium acetate, and glacial acetic acid were acquired from Meyer (Mexico City, Mexico). Ferric chloride hexahydrate, 2,4,6-tris (2-pyridyl)-s-triazine (TPTZ), hydrochloric acid, and ferrous chloride tetrahydrate were from JT Baker (Center Valley, PA, USA).

### 3.2. Sample Preparation

The plant *D. bicolor* was acquired in a market in Hidalgo, Mexico. The stems and leaves of the plant were dried at room temperature (~20 °C) for 20 days. Afterward, the dried sample was reduced in size (blender Oster BLSTEG7881R, Mexico) until made into a fine powder. It was stored at room temperature in airtight plastic jars until analysis.

### 3.3. Design of Experiments

Taking into account the number of factors to be analyzed in each extraction method, as well as information from the literature, two designs of experiments were applied to determine the best extraction conditions of antioxidant compounds and total phenolics in *D. bicolor*. For the conventional, microwave, and ultrasound-assisted extraction methods, the Box-Behnken design was applied; while for the French press, the Central Composite design was used. The conditions of experimentation (factors and levels) for the extraction process are presented in Table 3.

The control factors and selected levels were chosen by considering the normal conditions to prepare an infusion, which involves the use of water as extraction media. The chosen factors and levels depended on the analyzed extraction methods. For this selection, previous studies reported by other authors were also taken into account [33,54,55,56,57,58,59]. Due to the nature of each method used, it was not possible to replicate the conditions and factors selected. The design consisted of 15 experiments for Box-Behnken (DBB) and 13 experiments for Central Composite (DCC). All the experiments were done in triplicate.

Experimental data from the designs applied were analyzed using a response surface regression (Minitab v.17) fitted to a second-order polynomial model (Equation (1)).

(1) $$Y\;=\;\beta_{0}+\sum_{i\;=\;1}^{2}\beta_{i}x_{i}+\;\sum_{i\;=\;1}^{2}\beta_{ii}x_{i}^{2}+\sum_{i}*\sum_{j\;=\;i\;+\;1}\beta_{ij}x_{i}x_{j}$$

where: *Y* was the predicted response, *β*_0_ was the constant coefficient, *β*_1_, *β*_2_ were the linear coefficients, *β*_11_ and *β*_22_ were the quadratic coefficients, *β*_12_ was the cross-product coefficient, and *x*_1_…*x*_n_ were the independent variables. Contour plots were drawn out to show the simultaneous effect of the different factors studied on the experimental dependent parameters (antioxidant capacity and total phenolic content).

### 3.4. Extraction Methods

#### 3.4.1. Solid-Liquid

A conventional solid-liquid extraction was carried out by using a jacketed glass baker of 250 mL (Schott Duran^®^, Germany) on a hot plate stirring (Nuova Sarrer-Barnstead Thermolyne^®^ SP-131325, Waltham, MA, USA.) at 600 rpm and a recirculating bath (VWR^®^ MX07R-20, Randor, PA, USA.). To obtain the extracts, water was used as solvent under the conditions previously established in the experimental design: temperature (20, 55, and 90 °C), time (5, 15, and 25 min), and sample percentage (2, 6, and 10%) [33].

#### 3.4.2. Ultrasound-Assisted Extraction

The ultrasound-assisted extraction was carried out by following the methodology reported by Guo et al. [54] and Torres et al. [55] with some modifications. To prepare the extracts, the sample (2%, 6%, and 10%) was weighed in a beaker of 250 mL and the necessary volume of water was added. Afterward, the mixture was placed in an ultrasonic bath (Branson Ultrasonics^®^ 2510R-DTH, Connecticut, USA, 40 Hz of frequency) under the time (5, 15, and 25 min) and temperature (20, 45, and 70 °C) conditions previously established in the experimental design.

#### 3.4.3. Microwave

The extraction by microwave was performed according to the methodology described by Li, Skouroumounis, Elsey, and Taylor [56], and Routray and Orsat [57] with some modifications. A 750 W and 60 Hz microwave was used (LG^®^ MB-359ME, Korea). The mixture sample–water at different concentrations (2%, 6%, and 10%) was placed in a beaker of 250 mL and was submitted to the time (1, 1.5, and 2 min) and power conditions (20%, 30%, and 40%) previously established in the experimental design.

#### 3.4.4. French Press

To prepare the aqueous extracts, the methodology described by López et al. [58] and Rocha et al. [59] was followed. The corresponding amount of sample was weighed (0.34 and 11.6%) and it was placed in a French press (Bodum, Copenhagen, Denmark) with a capacity of 250 mL. Hot water at 90 °C was added and was left in contact with the sample for 0.76 to 9.2 min, according to the experimental design. Once the time of contact has passed, the plunger of the press was pushed down, and the overlying was recovered for posterior analysis.

Once the process of extraction of *D. bicolor* was finished, under the compared different methods, the obtained aqueous extracts were filtered to eliminate the remains of the plant that might cause interference. Finally, the response variables (total phenolic content and antioxidant activity) were analyzed.

### 3.5. Measurement of the Response Variables

#### 3.5.1. Antioxidant Activity by DPPH•

For this determination, the method suggested by Brand-Williams, Cuvelier, and Berset [60], was used with some modifications [22]. A calibration curve of 0 to 33 µM was prepared, on a basis of a standard solution of 1 mM Trolox in MeOH. To each standard solution, 2.9 mL of 0.1 mM DPPH• (2,2-diphenyl-1-picrylhydrazyl) in MeOH, were added. The absorbance was measured at 515 nm (spectrophotometer) using MeOH as blank. The antioxidant capacity was expressed as milligrams of equivalents of Trolox per 100 g of sample (mg ET/100 g).

#### 3.5.2. Ferric Reducing Antioxidant Power (FRAP)

This analysis was realized by using the FRAP technique of Benzie and Strain [61] with some modifications suggested by Chohan et al. [62]. The FRAP reagent was prepared from an acetate buffer (300 mM, pH 3.6), ferric chloride hexahydrate (20 mM), and 10 mM TPTZ (4,6-tripryridyl-s-triazine) prepared in 40 mM HCl. The three solutions were mixed in proportions of 10:1:1 (*v*/*v*/*v*). A calibration curve of 0 to 100 mM was prepared, through a standard solution of 40 mM ferrous chloride tetrahydrate (FeCl_2_•4H_2_O) in HCl. The absorbance was measured at 593 nm (spectrophotometer) using a blank only containing FRAP reagent. The obtained values were expressed as milligrams of ferrous ion equivalents per 100 g of sample (mg EFe^2+^/100 g).

#### 3.5.3. Total Phenolics by the Folin-Ciocalteu Method

For the quantification of total phenolics, a calibration curve was prepared in a concentration interval of 0 to 15 mg/L from a standard solution of 1000 mg/L gallic acid (GA). The corresponding volume was taken from each standard, 2 mL of Na_2_CO_3_ (7.5%) and 2 mL of Folin and Ciocalteu’s reagent (2 M) were added and it was diluted to 10 mL with distilled water. The absorbance was measured at 760 nm [22]. The results were expressed as milligrams of gallic acid equivalents per 100 g of sample (mg EAG/100 g). All determinations for variable responses were made in triplicate.

### 3.6. Optimization and Validation

The models were analyzed using the Minitab V. 17 software. A polynomial quadratic regression (Equation (1)) was used to determine the effects of the selected factors shown in Table 3. Linear, squared, and interaction coefficients were calculated.

#### Confirmatory Experiments

The optimum extraction point was determined through the statistical procedure applied. The desired goals for each variable and response were chosen. To validate the polynomial model, three replicates of aqueous extract of *D. bicolor* (confirmatory experiments) were prepared under the optimized levels of factors. The experimental values for each response were compared to the predicted data from the mathematical model.

## 4. Conclusions

The Box-Behnken and Central Composite experimental designs allow for identifying the influence of physical factors on the liberation of antioxidant and phenolic compounds from *Decatropis bicolor* in an aqueous medium. In general, the temperature was the determinant factor in the extraction of antioxidant compounds regardless of the method used. Besides, it was not necessary to use large amounts of the sample nor extended times of extraction to liberate higher concentrations of antioxidant and phenolic compounds. All the methods that were tested proved to be appropriate for the aqueous extraction of bioactive compounds; however, the French press was the most efficient method.

The values of antioxidant activity and phenolic compounds obtained from *Decatropis bicolor* by using the French press were superior even to those reported for other plant matrixes by using extraction methods with solvents such as methanol or ethanol. The results of this research show the potential of *D. bicolor* as a natural source of antioxidants. Further studies are necessary to quantify and identify the compounds responsible for the antioxidant properties of this plant widely used in Mexican traditional medicine.

## Figures and Tables

**Table 1:** Polynomials and correlation coefficients obtained from the experimental designs applied.

Extraction Method	Response Variable	R^2^	Polynomials
Conventional	Total phenolics	0.98	1449 + 7.88 A + 37.3 B − 206.5 C + 0.0045 A*A − 0.335 B*B + 11.41 C*C − 0.025 A*B + 0.028 A*C − 2.72B*C
	DPPH•	0.90	109 + 26.1 A + 59.1 B − 130.1 C − 0.1138 A*A −0.913 B*B + 11.97 C*C − 0.280 A*B − 1.259 A*C − 2.14B*C
	FRAP	0.99	1064.95 − 0.601 A − 2.957 B − 203.33 C + 0.00923 A*A + 0.0728 B*B + 11.485 C*C + 0.00581 A*B − 0.0431 A*C + 0.0460 B*C
Ultrasound	Total phenolics	0.95	4095 − 20.9 A − 97.1 B − 560.4 C + 0.386 A*A + 1.468 B*B + 45.49 C*C + 0.460 A*B − 1.796 A*C + 3.86 B*C
	DPPH•	0.91	497 + 5.4 A + 46.2 B − 49.5 C − 0.047 A*A − 1.207 B*B + 4.85 C*C + 0.210 A*B − 0.769 A*C − 1.88 B*C
	FRAP	0.98	928 + 1.11 A − 13.68 B − 177.2 C − 0.0090 A*A + 0.191 B*B + 10.27 C*C + 0.0885 A*B − 0.186 A*C + 0.579 B*C
Microwave	Total phenolics	0.99	5727 − 1.4 D − 942 B − 968.4 C − 0.266 D*D + 253 B*B + 47.96 C*C + 1.71 D*B + 2.25 D*C + 15.0 B*C
	DPPH•	0.99	1044 + 36.6 D − 245 B − 135.3 C − 0.364 D*D + 156 B*B + 8.70 C*C − 1.24 D*B − 1.474 D*C − 19.8 B*C
	FRAP	0.99	868 + 13.25 D + 131 B − 221.7 C − 0.228 D*D − 59.9 B*B + 9.980 C*C − 2.07 D*B + 0.496 D*C + 12.81 B*C
French press	Total phenolics	0.86	7186 + 407 B − 1815 C − 42.6 B*B + 105.8 C*C+ 2.3 B*C
	DPPH•	0.88	1506 + 107 B − 288.5 C − 7.57 B*B + 16.41 C*C − 2.38 B*C
	FRAP	0.88	1467 + 68 B − 363.4 C − 7.6 B*B + 20.86 C*C + 1.0 B*C

A: Temperature, B: Time, C: Sample, D: Power.

**Table 2:** Results of the antioxidant activity and total phenolic of the aqueous extracts of *D. bicolor* obtained based on the experimental design.

Extraction Methods	Conditions			mg EGA/100 g	mg ET/100 g	mg EFe^2+^/100 g
	T (°C)	t (min)	Sample (g/100 g)	Mean ± SD	Mean ± SD	Mean ± SD
Conventional extraction	90	5	6	1480.8 ± 5.5	532.8 ±1.4	247.4 ± 1.8
	55	25	10	1050.0 ± 2.5	522.7 ±0.4	142.4 ± 0.1
	90	15	10	1380.2 ± 5.2	301.5 ±1.2	151.2 ± 1.4
	55	25	2	2139.0 ± 5.7	1234.6 ±2.8	679.6 ± 6.3
	55	15	6	1296.3 ± 4.8	687.3 ±2.9	219.8 ± 1.3
	20	25	6	1073.0 ± 4.4	581.0 ±1.0	225.8 ± 2.9
	55	15	6	1296.3 ± 1.0	691.0 ±0.8	219.5 ± 1.4
	55	5	10	969.3 ± 1.1	516.1 ±0.7	138.9 ± 0.8
	20	15	2	1596.3 ± 2.9	828.9 ±2.8	666.5 ± 1.6
	90	15	2	2232.2 ± 2.9	1511.5 ±5.7	691.6 ± 11.1
	90	25	6	1547.0 ± 4.8	295.5 ±1.0	244.5 ± 5.5
	55	5	2	1622.8 ± 5.7	885.0 ±2.3	683.4 ± 8.8
	55	15	6	1296.1 ± 2.8	689.9 ±2.4	220.8 ± 1.7
	20	5	6	972.5 ± 1.0	424.8 ±1.6	236.8 ± 2.0
	20	15	10	739.6 ± 5.2	314.5 ±3.6	144.4 ± 1.6
Ultrasound- assisted extraction	45	15	6	1257.7 ± 5.7	705.4 ± 1.9	166.6 ± 3.1
	45	15	6	1258.4 ± 8.6	708.7 ± 9.5	165.0 ± 1.4
	45	15	6	1261.3 ± 6.1	705.4 ± 1.9	165.6 ± 2.5
	45	5	10	2071.7 ± 1.1	508.7 ± 3.1	109.9 ± 1.1
	70	15	2	2971.0 ± 2.3	1215.8 ± 9.6	553.2 ± 2.4
	70	15	10	2187.6 ± 5.2	506.7 ± 0.7	135.2 ± 1.4
	20	15	10	1845.1 ± 0.9	447.6 ± 1.8	132.8 ± 2.5
	70	5	6	1684.6 ± 1.9	292.4 ± 2.2	149.9 ± 1.1
	45	5	2	2556.4 ± 4.7	669.4 ± 8.6	633.6 ± 2.4
	45	25	10	2019.9 ± 5.3	506.5 ± 3.4	111.1 ± 1.1
	20	5	6	1616.8± 9.6	643.4 ± 10.1	182.9 ± 0.6
	45	25	2	1887.0 ± 5.7	968.7 ± 5.3	542.2 ± 3.3
	70	25	6	1908.1 ±1.9	574.5 ± 5.1	219.8 ± 2.2
	20	15	2	1910.0 ± 2.3	848.9 ± 7.8	476.5 ± 5.8
	20	25	6	1380.1 ± 4.8	715.5 ± 7.7	164.3 ± 1.0
Extraction with microwave	30 *	2	2	3152.7 ± 2.4	1514.8 ± 9.0	631.9 ± 1.7
	20 *	1.5	10	693.6 ± 5.6	519.4 ± 0.5	134.4 ± 1.3
	20 *	1	6	1135.1 ± 3.9	748.9 ± 1.0	212.1 ± 3.9
	40 *	1.5	10	715.6 ± 6.2	522.0 ± 0.5	129.0 ± 1.3
	20 *	2	6	1103.0 ± 4.7	812.5 ± 1.3	213.8 ± 0.7
	30 *	2	10	712.3 ± 3.7	520.6 ± 0.8	130.8 ± 1.1
	40 *	1.5	2	2879.0 ± 8.6	1401.6 ± 5.2	623.1 ± 5.8
	40 *	2	6	1226.6 ± 5.8	841.5 ± 3.9	215.1 ± 0.8
	30 *	1.5	6	1132.8 ± 5.8	812.6 ± 2.6	261.0 ± 0.6
	30 *	1.5	6	1139.2 ± 4.4	771.2 ± 1.7	263.2 ± 1.8
	30 *	1.5	6	1134.9 ± 0.3	812.6 ± 2.6	261.0 ± 0.6
	30 *	1	2	3280.4 ± 5.8	1354.0 ± 1.2	733.3 ± 3.6
	20 *	1.5	2	3217.2 ± 2.9	1163.2 ± 5.5	707.8 ± 1.8
	40 *	1	6	1224.5 ± 8.6	802.7 ± 4.9	254.9 ± 0.4
	30 *	1	10	719.6 ± 2.8	518.0 ± 0.3	129.7 ± 0.9
French press	90	0.76	6	1166.8 ± 2.4	557.4 ± 2.8	208.9 ± 2.9
	90	5	11.7	652.3 ± 5.5	434.9 ± 1.4	110.8 ± 1.0
	90	5	6	1153.7 ± 3.4	629.0 ± 1.4	218.3 ± 1.8
	90	2	2	2724.8 ± 10.3	861.8 ± 6.5	601.7 ± 3.6
	90	5	6	1112.5 ± 4.1	590.7 ± 2.3	211.7 ± 1.6
	90	5	0.34	9929.1 ± 41.1	2128.2 ± 12.0	1920.4 ± 4.8
	90	9.24	6	1111.6 ± 2.8	682.8 ± 2.2	213.5 ± 0.5
	90	5	6	1127.9 ± 2.8	679.0 ± 2.2	220.5 ± 0.8
	90	8	2	2647.4 ± 9.4	1033.8 ± 7.0	550.1 ± 4.9
	90	8	10	715.3 ± 4.6	519.1 ± 0.6	128.2 ± 0.5
	90	5	6	1141.0 ± 4.1	663.7 ± 3.5	219.4 ± 2.0
	90	2	10	684.6 ± 2.4	461.2 ± 1.4	130.3 ± 0.4
	90	5	6	1191.3 ± 3.8	639.9 ± 1.8	217.9 ± 1.1

* Power.

**Table 3:** Conditions of the experimental designs for the extraction of antioxidant compounds and total phenolics of *D. bicolor* in an aqueous medium.

**Conventional Extraction**		**Selected Levels**		
Control factors		−1	0	1
A	Temperature (°C)	20	55	90
B	Time (min)	5	15	25
C	Sample (g/100 g)	2	6	10
**Ultrasound-assisted extraction**		**Selected levels**		
Control factors		−1	0	1
A	Temperature (°C)	20	45	70
B	Time (min)	5	15	25
C	Sample (g/100 g)	2	6	10
**Extraction with microwave**		**Selected levels**		
Control factors		−1	0	1
A	Power (%)	20	30	40
B	Time (min)	1	1.5	2
C	Sample (g/100 g)	2	6	10
**French press**		**Selected levels**		
A	Time (min)		0.76	9.2
B	Sample (g/100 g)		0.34	11.6

## Data Availability

The data presented in this study are available in this article.

## Sample Availability

Not available.
